# Draft Genome Sequence of Paenibacillus sonchi IIRRBNF1, a Nitrogen-Fixing and Plant Growth-Promoting Bacterium Isolated from Rice Rhizosphere

**DOI:** 10.1128/mra.00126-22

**Published:** 2022-04-06

**Authors:** Sonth Bandeppa, Amol S. Phule, Kalyani M. Barbadikar, Rajani Govindannagari, Prasad Babu M. B. B, Venkat Prasad Babu Kavuru, Pranab Kumar Mandal, Raman Meenakshi Sundaram, Latha P. Chandran

**Affiliations:** a ICAR-Indian Institute of Rice Research, Hyderabad, Telangana, India; b ICAR-National Institute for Plant Biotechnology, New Delhi, Delhi, India; University of Delaware

## Abstract

Paenibacillus sonchi IIRRBNF1 is a rice-rhizospheric, endospore-forming, Gram-positive, plant growth-promoting rhizobacterium. Here, we report the draft genome sequence of Paenibacillus sonchi IIRRBNF1, which consists of an∼7.3-Mb (7,323,556-bp) genome with 6,271 coding sequences (CDSs), 13 rRNAs, and 67 tRNAs. The genome reveals the presence of a nitrogen-fixing gene cluster and genes associated with multiple plant growth-promoting traits.

## ANNOUNCEMENT

Paenibacilli are aerobic endospore-forming bacteria, present ubiquitously in agricultural soils. Plant- and soil-associated paenibacilli are known to promote plant growth by production of phytohormones like indole-3-acetic acid, solubilizing inaccessible plant nutrients and fixing atmospheric nitrogen (1). Paenibacillus sonchi IIRRBNF1 was isolated from the rhizosphere of rice grown in the Nalgonda district (17°3′36″N, 79°18′0″E) of Telangana, India, using a nitrogen-free medium. Briefly, a serially diluted rhizosphere soil sample was plated on nitrogen free Rennie’s medium (2) and incubated at 28°C for 7 days. Bacterial colonies with unique morphologies which appeared on the petri dishes after incubation were selected, purified, and characterized, and an isolate, IIRR F4, was identified by using 16S rRNA gene sequencing (3) and renamed Paenibacillus sonchi IIRRBNF1 (GenBank accession number MZ905164). Here, we present the draft genome sequence of Paenibacillus sonchi IIRRBNF1 and report on the genes potentially involved in nitrogen fixation and plant growth promotion.

A log-phase culture of Paenibacillus sonchi IIRRBNF1 grown in Rennie’s broth at 28°C was used for DNA extraction. A standard protocol was used to extract the genomic DNA of the isolate Paenibacillus sonchi IIRRBNF1 (4), and the quality and quantity of the DNA were checked using a NanoDrop ND-1000 instrument, followed by separation on a 0.8% agarose gel. Paired-end sequencing libraries were constructed using a TruSeq Nano DNA preparation kit by following the manufacturer’s protocols (Illumina, CA), and the quality and quantity of libraries were assessed on a 4200 Tape Station system (Agilent Technologies, CA). Genome sequencing was performed with the Illumina NextSeq 500 (2 × 150-bp paired-end sequencing) platform. Trimmomatic v0.38 (sliding window, 10:20; leading, 20 and trailing, 20) was used to remove ambiguous reads, low-quality sequences, and adapter sequences (5), and a total of 5,486,488 high-quality sequence reads (∼1.58 Gb) was generated. The high-quality paired-end reads were *de novo* assembled using the SPAdes (v3.13.0) genome assembler (6), followed by genome refinement using GFinisher (7). The draft genome consisted of 66 scaffolds (minimum scaffold size of 210 bp and maximum scaffold size of 5,31,015 bp) with 7,323,556 bases (∼7.3 Mb) and an *N*_50_ of 256,778 bp, with 230× genome coverage and a GC content of 50.9%. A total of 6,356 genes were annotated using the NCBI Prokaryotic Genome Annotation Pipeline (PGAP) v5.2 (8, 9). The genome has 6,271 coding sequences (CDSs), 13 rRNAs, 67 tRNAs, and 1 transfer-messenger RNA (tmRNA). The gene ontology (GO) annotation of the predicted genes was determined by using Blast2GO (10) and KEGG GENES database using KAAS (11). Default parameters were used for all software unless mentioned otherwise.

The Paenibacillus sonchi IIRRBNF1 genome carries the *nif* gene cluster, *viz.*, *nifB*, *nifH*, *nifD*, *nifK*, *nifE*, *nifN*, *nifU*, *nifX*, and *hesA*. In addition, the *P. sonchi* IIRRBNF1 genome also harbors the genes encoding plant growth promotion traits, including an ability to improve nutrient availability (*pqq* and *phoP*), plant hormone synthesis (*trpC*), antimicrobial activity (*phzF*), and siderophore production (*pvdO* and *fhuC*). The genome also possesses genes involved in quorum sensing, oxidative stress resistance, abiotic stress tolerance, and CRISPR arrays.

The average nucleotide identity based on BLAST+ (ANIb) determined using JSpecies software (12) showed that *P. sonchi* IIRRBNF1 and *P. sonchi* LMG 24727^T^ (13) had an ANIb value of 98.28%, indicating that both bacteria belong to the same species.

Thus, an in-depth analysis of the *P. sonchi* IIRRBNF1 genome will provide deeper insights for understanding the nitrogen fixation pathways and underlying mechanisms that control the bacterial ability to provide fitness advantages to the plant.

### Data availability.

The draft genome sequence of Paenibacillus sonchi IIRRBNF1 has been deposited in NCBI GenBank with the accession number JAIFKC000000000 and the SRA accession number SRR17868980. The BioProject and BioSample accession numbers associated with the genome sequence are PRJNA753291 and SAMN20693057, respectively.
